# Gingipains protect *Porphyromonas gingivalis* from macrophage-mediated phagocytic clearance

**DOI:** 10.1371/journal.ppat.1012821

**Published:** 2025-01-21

**Authors:** Magdalena Widziolek, Anna Mieszkowska, Magdalena Marcinkowska, Bartlomiej Salamaga, Justyna Folkert, Krzysztof Rakus, Magdalena Chadzinska, Jan Potempa, Graham P. Stafford, Tomasz K. Prajsnar, Craig Murdoch

**Affiliations:** 1 Institute of Zoology and Biomedical Research, Jagiellonian University, Kraków, Poland; 2 School of Clinical Dentistry, University of Sheffield, Sheffield, United Kingdom; 3 Doctoral School of Exact and Natural Sciences, Jagiellonian University, Kraków, Poland; 4 School of Biosciences, University of Sheffield, Sheffield, United Kingdom; 5 Department of Microbiology, Faculty of Biochemistry, Biophysics and Biotechnology, Jagiellonian University, Krakow, Poland; 6 Department of Oral Immunity and Infectious Diseases, University of Louisville School of Dentistry, Kentucky, United States of America; 7 The Florey Institute, University of Sheffield, Sheffield, United Kingdom; University of Massachusetts Medical School, UNITED STATES OF AMERICA

## Abstract

*Porphyromonas gingivalis (Pg)* is a keystone pathogen in periodontitis, a highly prevalent disease manifested by chronic inflammation of the periodontium, alveolar bone resorption and tooth loss. During periodontitis pathobionts such as *Pg* can enter the bloodstream and growing evidence correlates periodontitis with increased risk of cardiovascular and neurodegenerative diseases. However, the mechanism by which immune cells respond to *Pg* challenge *in vivo* remains elusive. *Pg* produce aggressive proteolytic virulence factors termed gingipains which not only provide nutrients necessary for bacterial growth, but also subvert the host immune response, facilitating bacterial survival. Using transgenic zebrafish with fluorescently labelled macrophages and neutrophils, the role of gingipains in bacterial survival and interaction with phagocytes during systemic and local infection was examined. In contrast to the wild-type (W83) *Pg*, isogenic gingipain-null (*ΔK/R-ab)* or wild-type *Pg* treated with gingipain inhibitors caused less zebrafish mortality, bacteria were rapidly phagocytosed, acidified in phagosomes and eradicated when systemically injected, showing that gingipains are instrumental in preventing phagocytosis and intracellular killing of *Pg* by innate immune cells. Moreover, *Pg* were predominantly phagocytosed by macrophages, and gingipain depletion/inactivation increased bacterial phagocytosis when bacteria were injected either systemically or locally in the otic vesicle, with less bacteria internalised by neutrophils. This phenomenon was *Pg*-specific as *Fusobacterium nucleatum* caused neutrophil recruitment that then effectively phagocytosed these bacteria. These data demonstrate the important role of phagocytes, especially macrophages, in combating *Pg* infection and highlight the crucial protective role of gingipains in subverting the innate immune response. This study also emphasizes the advantages of using zebrafish to study interactions of *Pg* with phagocytes *in vivo* in real-time, providing a valuable experimental system for testing new therapeutic strategies aimed at reducing periodontal-associated systemic or neurodegenerative disease.

## Author summary

*Porphyromonas gingivalis (Pg)* is bacterium found within the oral cavity, particularly the biofilm of dental plaque. *Pg*, along with other pathobionts, are responsible for periodontitis where bacterial-mediated chronic inflammation leads to destruction of tooth supporting structures and consequently tooth loss. At these oral inflammatory sites *Pg* can enter the bloodstream and has been found in atherosclerotic plaques within arteries. There is now substantial evidence that this bacterium is associated with cardiovascular and neurodegenerative disease. Here, we used transgenic zebrafish where macrophages and neutrophils are fluorescently labelled to explore how *Pg* interacts with phagocytes, and the mechanism by which this bacterium escapes immune surveillance. We found that macrophages phagocytose and kill *Pg* more effectively than neutrophils, both in the circulation and within tissue. However, bacterial eradication was substantially inhibited by *Pg*-specific proteases termed gingipains that markedly reduced both phagocytosis and bacterial killing within macrophages. Our study provides new insights into the host innate immune response toward *Pg* and identify bacterial virulence proteins that could be targets for therapeutic intervention for periodontal-mediated cardiovascular or neurodegenerative disease.

## Introduction

*Porphyromonas gingivalis (Pg)* is a Gram-negative oral-dwelling bacterium commonly found within the anaerobic environment of sub-gingival dental plaque. Here, *Pg*, along with other pathobionts, form a biofilm that is ultimately responsible for the development of periodontitis where bacterial products along with bacterial-mediated chronic inflammation leads to destruction of tooth supportive structures and consequently tooth loss [1,2]. In this regard, *Pg* is often termed a keystone pathogen in periodontal disease [3].

Initially, *Pg* interacts with gingival epithelial cells where it can persist intracellularly, driving disease chronicity [4]. As the disease progresses, phagocytes are recruited to the site of infection and are activated by microbial virulence factors. Abundant neutrophils are found within gingival tissues and the gingival crevicular fluid of periodontal patients, but despite being equipped with many antibacterial weapons, these phagocytes often fail to efficiently control infection [5–7]. In addition to neutrophils, macrophages are also abundant in the inflamed periodontium [8]. These cells play a pivotal role in inducing and regulating the immune response against invading microbes by phagocytosis of bacteria, antigen presentation and secretion of cytokines that co-ordinate the innate and adaptive immune responses.

*Pg* has developed several approaches to persist within host tissues through the secretion of a range of virulence factors, which include cysteine proteases termed gingipains that are either lysine-specific (Kgp) or arginine-specific (Rgp A and B). There is substantial evidence that gingipains are fundamental for *Pg*-mediated pathogenesis as well as in the maturation of other virulence factors [9–11]. Gingipains play a critical role at different stages of periodontitis progression. In early disease, they participate in nutrient acquisition, biofilm colonization and bacterial survival, while in later stages they cleave host proteins leading to impaired bacterial recognition, phagocytosis and tissue repair and dysregulation of the immune response while mediating host tissue destruction and bacterial dissemination [12–14].

Recently, evidence has shown a correlation between periodontitis and conditions such as cardiovascular disease [15,16] and neurodegenerative disorders [17,18]. Periodontal pathobionts such as *Pg* can enter the circulation at diseased periodontal sites and be disseminated systemically. Their presence has been detected in vascular tissues [19]. Although the precise mechanism by which bacteria enter the bloodstream is poorly understood, their presence in the blood and interaction with circulating phagocytes is crucial for disease progression.

*Pg*-mediated dysregulation of phagocyte immune responses may cause uncontrolled production of pro-inflammatory mediators into the circulation leading to vascular inflammation and endothelial dysfunction [20]. Moreover, once phagocytosed, *Pg* may survive intracellularly within phagocytes, travel around the body and be deposited at distant sites in a ‘Trojan-horse-like’ mechanism as phagocytes leave or enter the circulation [21]. It is therefore necessary to understand the kinetics and effects of phagocyte-*Pg* interaction, preferably in an *in vivo* setting.

Recently, we showed that *Pg* caused vascular damage in a gingipain-dependent manner by cleavage of endothelial cell surface adhesion molecules in a zebrafish (*Danio rerio*) larvae systemic infection model [22,23]. However, the interaction of *Pg* with phagocytes in this model has not been investigated previously. Here, we use the *Pg*-zebrafish larvae infection model to show that gingipains play a protective role in their interactions with phagocytes *in vivo*, enhancing *Pg* survival. We showed that gingipain-depleted bacteria and wild-type *Pg* treated with gingipain inhibitors are rapidly phagocytosed in contrast to the wild-type bacteria producing active gingipains. In addition, for the first time, we show the crucial role of macrophages in clearance of *Pg in vivo*.

## Results

### Gingipains are important for *Pg* survival in zebrafish larvae

**Fig 1:**
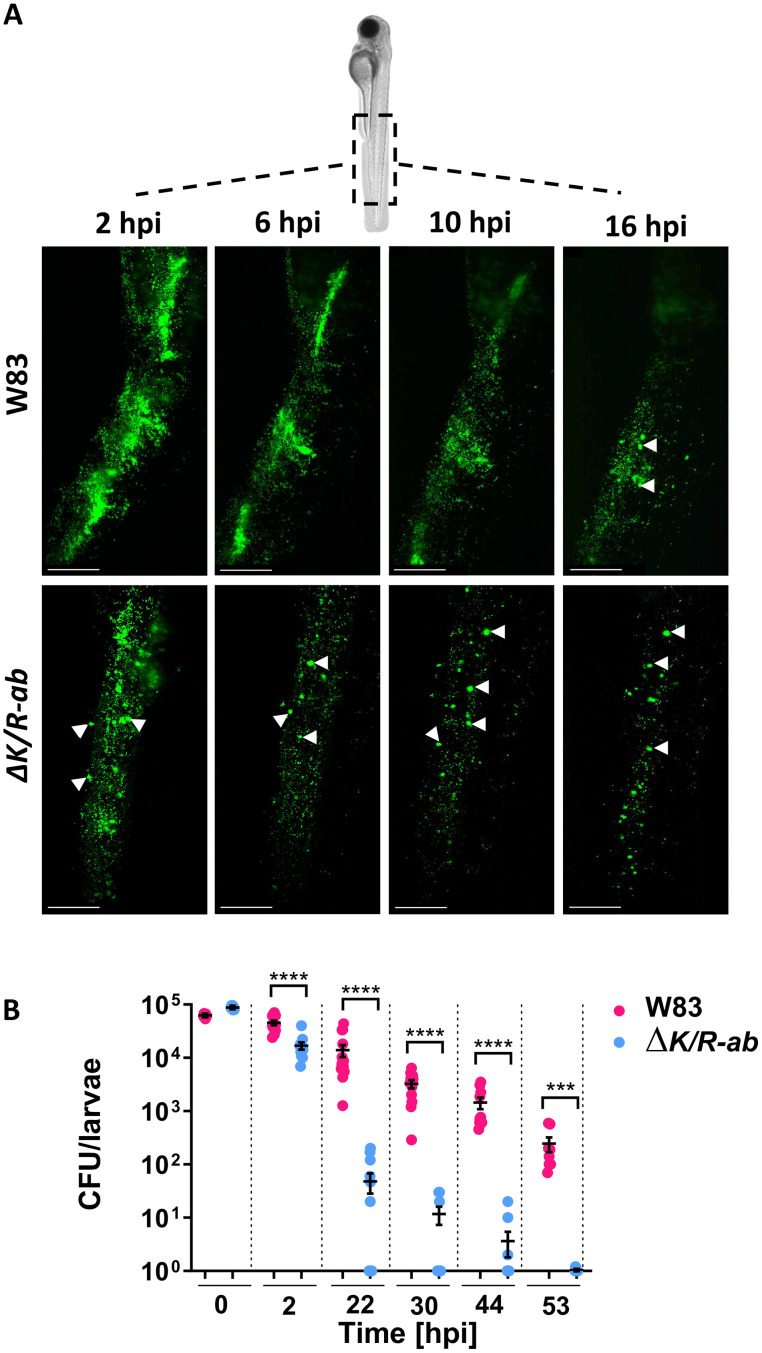
Wild-type *Porphyromonas gingivalis (Pg)* W83 strain displays greater levels of survival in systemically infected zebrafish larvae compared to gingipain-null *ΔK/R-ab* mutant. Zebrafish embryos were infected systemically at 30 hpf with fluorescein-SE-labelled wild-type *Pg* W83 or the gingipain-null mutant *ΔK/Rab*. PBS was injected into control larvae. (A) Representative images of real-time light-sheet microscopy over 16 h showing the differences in the course of bacterial eradication. White arrowheads point to *Pg* localised in puncta. Scale bar = 100 μm. (B) Zebrafish larvae were infected with *Pg* W83 or *ΔK/R-ab*, and larvae were homogenized at indicated time post-infection (hpi), serially diluted and number of recovered bacteria were enumerated on ABA plates. CFU–colony forming unit. Data are presented as means ± SEM. Each dot represents the number of bacteria recovered from an individual larva. Statistical significance was analysed by Mann-Whitney test, *** p≤0.001, **** p ≤ 0.0001.

Previously, we observed high mortality rates of zebrafish larvae following systemic infection with wild-type (W83) *Pg* strain compared to the gingipain-null mutant, *ΔK/R-ab* [22]. Therefore, the ability of these bacteria to survive within the zebrafish host was examined. We observed that the fluorescent signal from fluorescein-SE-labelled *ΔK/R-ab Pg* cells decreased much faster than for labelled-W83, suggesting that the *ΔK/R-ab* mutant was cleared from zebrafish noticeably quicker than wild-type bacteria over 16 hours (Fig 1A). In addition, *ΔK/R-ab Pg* was observed clustered within large puncta within the zebrafish tail region, rather than dispersed throughout the tail as observed in W83 infected zebrafish, suggesting that gingipain-null *Pg* were being rapidly internalised by cells within zebrafish (Fig 1A). Survival of *Pg* W83 and *ΔK/R-ab* was further evaluated up to 53 hours post-infection (hpi) by colony forming unit (CFU) count following larval homogenisation. The levels of *ΔK/R-ab* significantly decreased in a time-dependent manner compared to larvae infected with *Pg* W83 (Fig 1B; p ≤ 0.0001). *Pg* W83 was able to survive within the larvae for at least 53 hpi compared to the *ΔK/R-ab*, where total clearance in some larvae started from 22 hpi and the mutant bacteria were completely cleared in all larvae by 53 hpi (Fig 1B; p ≤ 0.01). These data indicate that gingipains significantly influence the ability of zebrafish larvae to clear *Pg* following systemic infection.

### Myeloid cell depletion increases the mortality of *Pg*-infected zebrafish larvae

**Fig 2:**
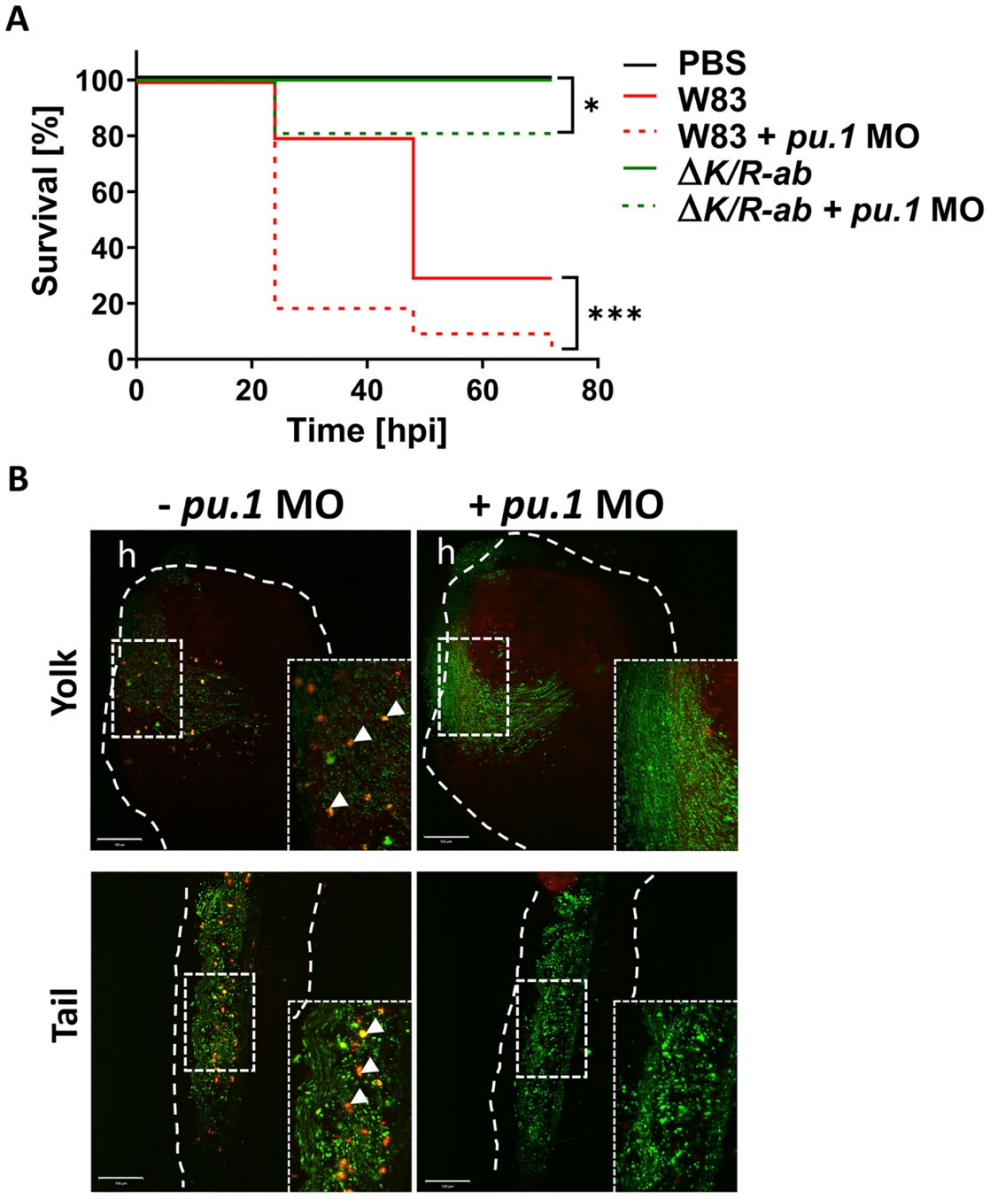
Myeloid cell-depleted zebrafish larvae are more susceptible to *Porphyromonas gingivalis (Pg)* infection. (A) Kaplan-Meyer survival plot of *pu*.*1* MO-treated or PBS-injected control zebrafish larvae upon systemic infection with the wild-type *Pg* W83 or *ΔK/R-ab*. Survival plots were analysed by log-rank test.*p≤0.05,***p≤0.001; hpi- hours post infection. (B) Representative images of the yolk and tail region of *pu*.*1* MO-treated and untreated zebrafish larvae 2 hpi with fluorescein-SE (green) and pHrodo (red) dual-stained *ΔK/R-ab*. The untreated larvae display orange dots (white arrows) representing phagocytosed (phagosome acidified) *ΔK/R-ab*. Phagocytosed *ΔK/R-ab* were not detected in *pu*.*1* MO due to removal of myeloid cells. White dashed lines indicate yolk or tail areas. Boxes show zoomed regions of the images. h-heart. Scale bar = 100 μm.

To evaluate the role of phagocytes during *Pg* systemic infection, *pu*.*1* knockdown zebrafish larvae devoid of all myeloid lineage cells were infected with *Pg* W83 or *ΔK/R-ab*. Kaplan-Meyer survival analysis revealed that *pu*.*1* larvae were more susceptible to infection with *Pg* W83 (4% survival; p ≤ 0.001) and *ΔK/R-*ab (80% survival; p ≤ 0.05) when compared to their myeloid-competent counterparts (30% for W83 and 100% survival for *ΔK/R-*ab, respectively; Fig 2A). Thus, phagocyte-depleted zebrafish are much more susceptible to wild-type *Pg* W83 infection compared to immunocompetent zebrafish. P*u*.*1* morpholino-treated or control larvae were also infected with dual-labelled pHrodo (a pH responsive dye that fluoresces at low pH—i.e., only within acidified phagosomes) and fluorescein-SE (all *Pg* green) *ΔK/R-*ab. The abundant presence of pHrodo-positive bacteria, representing bacteria within the acidified phagosomes of phagocytes, were evident for control larvae, whereas this was not observed in *pu*.*1* morphants (Fig 2B). These data not only confirm the essential role of phagocytes in clearing *Pg* systemic infection but also demonstrate the important role of gingipains in bacterial pathogenesis.

### Gingipains prevent *Pg* acidification within the phagosomes of zebrafish myeloid cells

**Fig 3:**
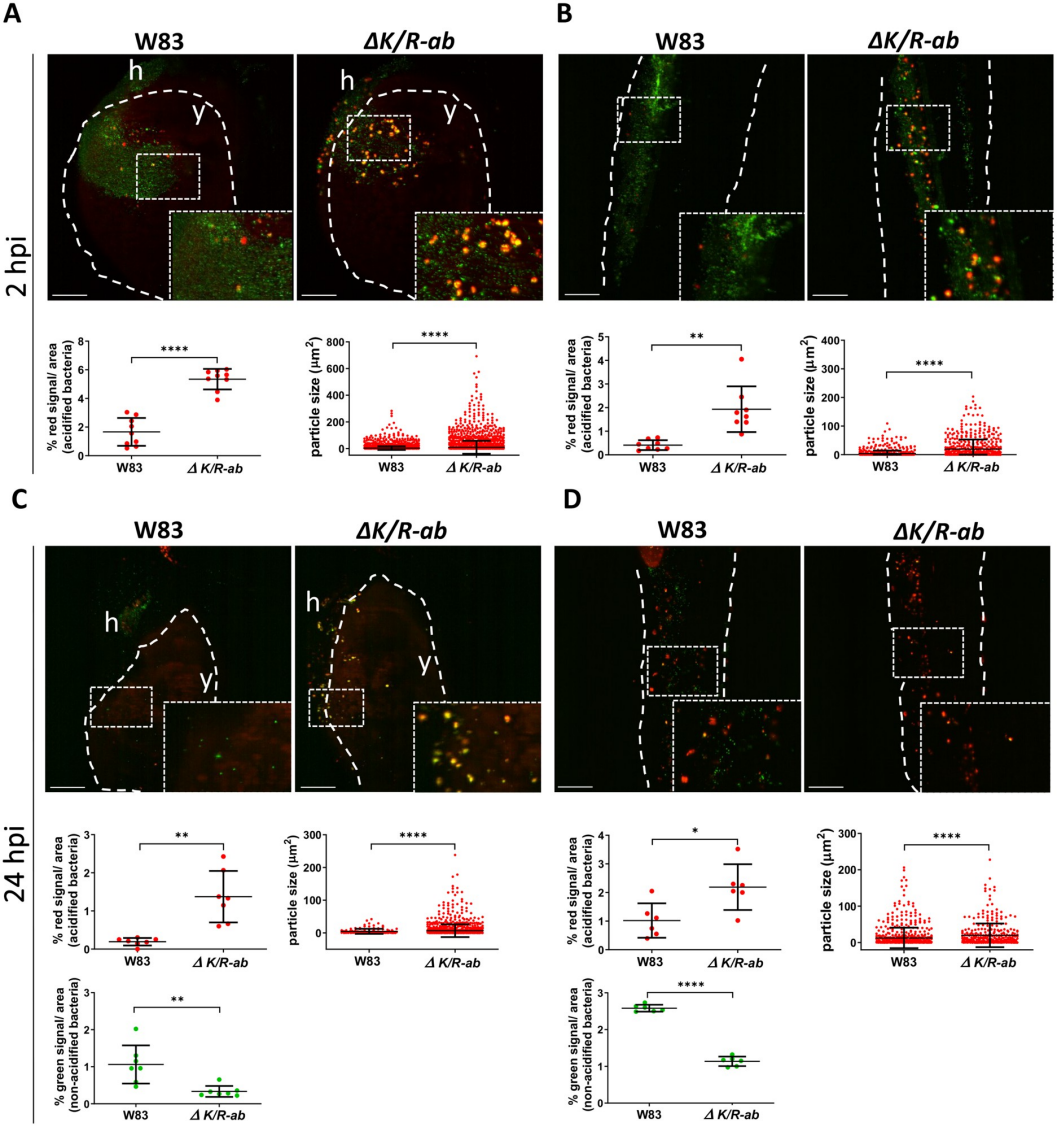
Gingipains prevent phagosome acidification of internalized *Porphyromonas gingivalis (Pg)*. Real-time in vivo examination of the acidification of the wild-type *Pg* W83 and *ΔK/R-ab*. *Pg* were stained with fluorescein-SE (green) and pHrodo (red) and zebrafish larvae (30 hpf) were infected into the Duct of Cuvier. Representative images along with quantification of the phagosome-acidified bacteria within selected area and size of acidified bacterial clusters in the yolk (A and C) or the tail regions (B and D) at 2 hpi (A and B) or 24 hpi (C and D). White dashed lines indicate yolk or tail areas. Boxes show zoomed regions of the images. y = yolk, h = heart. Scale bar = 100 μm. Data presented are means ± SD. Each dot represents signal quantified from a single zebrafish larva. Statistical significance was analysed by unpaired t-test with Welch’s correction or Mann-Whitney test where required. *≤0.05, **p≤0.01, ****p≤0.0001.

The role of gingipains in bacterial killing by phagocytes was next examined at 2 and 24 hpi using dual-labelled pHrodo/fluorescein-SE *Pg*. At 2 hpi larvae infected with *ΔK/R-ab Pg* displayed 3-fold greater pHrodo signal/image area from bacteria within the acidified phagosomes compared to *Pg* W83 (5.4% ± 0.7% compared to 1.7% ± 1.0% respectively; p ≤ 0.0001) analysed in the common cardinal vein in the yolk region (Fig 3A). The size of the pHrodo-positive particles was also 3-fold larger for *ΔK/R-ab* compared to *Pg* W83 (9.2 μm ± 48.5 μm compared to 3.0 μm ± 14.1μm respectively; p ≤ 0.0001; Fig 3A), suggesting that the phagocytes engulfed many *ΔK/R-ab* whereas phagocytosis of wild-type *Pg* W83 was rare. Similar findings were observed in the tail region of these zebrafish larvae regarding the increased *ΔK/R-ab* acidification (p ≤ 0.01) and the size of the acidified bacterial clusters (p ≤ 0.0001; Fig 3B) compared to *Pg* W83. At 24 hpi increased levels of acidified bacteria was still observed for *ΔK/R-ab* compared to the wild-type W83 *Pg*, in both the yolk (7-fold increase, 1.4% ± 0.7% versus 0.2% ± 0.1%; respectively; p ≤ 0.01) and tail regions (2-fold increase, 2.2% ± 0.8% versus 1.0% ± 0.6%; p ≤ 0.05; Fig 3C and 3D). This increase in pHrodo-positive signal was associated with a concurrent decrease in overall bacterial load (loss of green fluorescence) that was particularly evident in the tail region at 24 hpi (Fig 3D). There was a 3-fold decrease for the *ΔK/R -ab* (0.3% ± 0.1%) compared to the wild-type W83 (1.0% ± 0.3%) in the yolk region (p ≤ 0.01), and a 2-fold decrease in the tail region (1.1% ± 0.1% and 2.6% ± 0.1% respectively, p ≤ 0.0001).

Taken together, these data indicate not only that *ΔK/R-ab Pg* are acidified rapidly and in large numbers but also that gingipains may prevent *Pg* from phagocytic uptake and/or phagosome acidification, and consequently bacterial killing. For both wild-type and the *ΔK/R-ab*, some of the fluorescein-SE-labelled bacteria were observed as bigger clusters, plausibly within phagocytes, suggesting that, although internalized, these bacteria were not yet within acidified phagosomes (Fig 3A–3D). However, we cannot exclude that, some of these bacteria could be located at the phagocyte cell surface.

**Fig 4:**
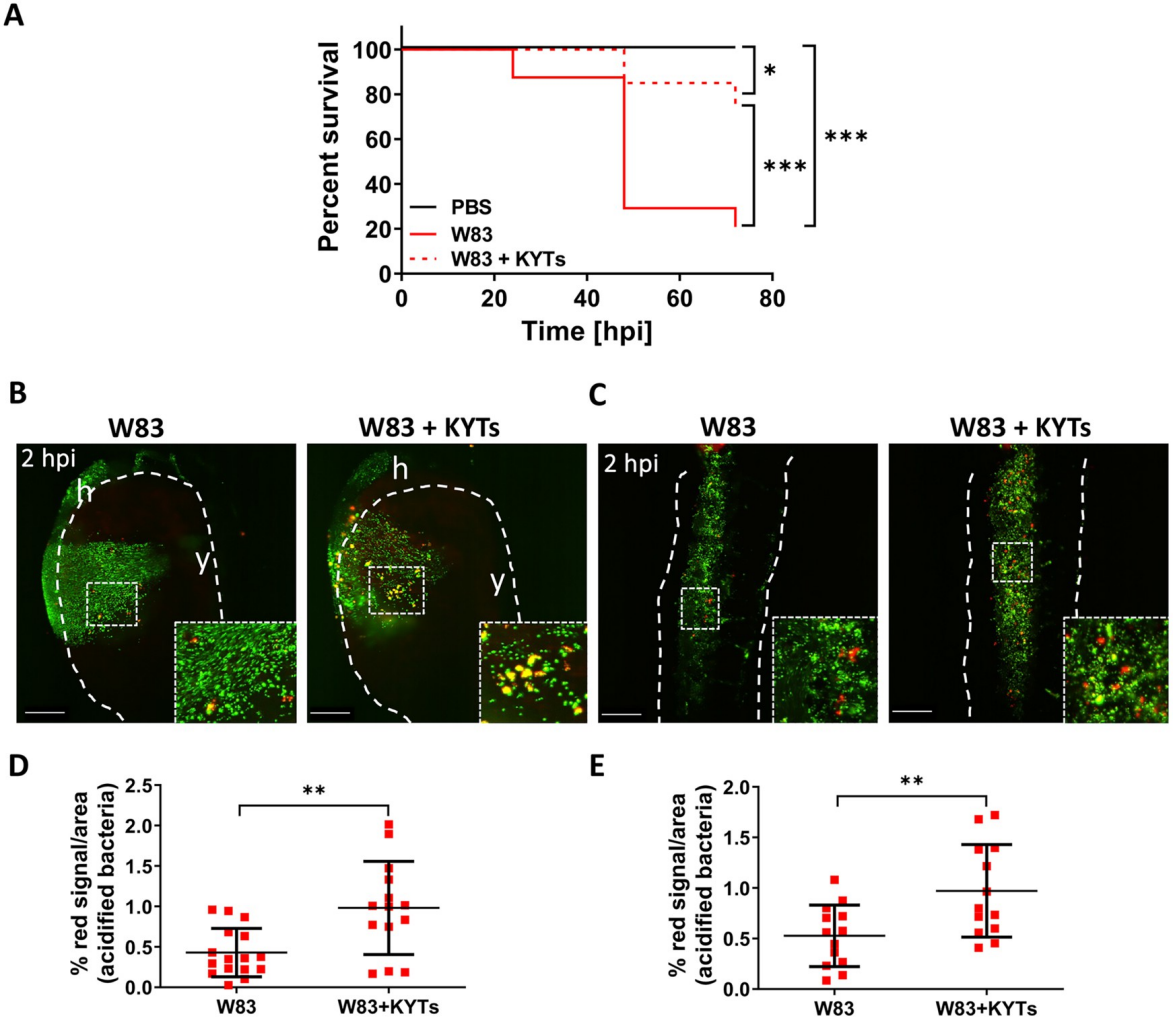
Inactivation of gingipains by KYT inhibitors increases larvae survival and increases phagocytosis of wild-type W83 *Porphyromonas gingivalis (Pg)*. (A) Kaplan-Meier survival plot of zebrafish larvae (30 hpf) infected with wild-type *Pg* W83 pre-treated or untreated with KYT inhibitors. Comparisons between survival curves were made using the log rank test. *p≤0.05,***p≤0.001; hpi- hours post infection. Real-time analysis of the acidification process during phagocytosis of the *Pg* W83 treated/untreated with KYT inhibitors in the yolk (B) and tail (C) regions 2 hpi. *Pg* were stained with fluorescein-SE (green) and pHrodo (red). White dashed lines indicate yolk or tail areas. Boxes show zoomed regions of the images. y-yolk, h-heart. Scale bar = 100 μm. Quantified data from the yolk (D) and the tail (E) regions. Data are presented as means ± SD. Each dot represents signal quantified from a single larva. Statistical significance was analysed by unpaired t-test with Welch’s correction. **p≤0.01.

To confirm that *Pg* gingipain activity directly interferes with phagocytosis, wild-type W83 *Pg* were pre-treated with gingipain inhibitors (KYTs) and double-labelled with fluorescein-SE and pHrodo, before levels of phagocytosis were measured at 2 hpi. Systemic infection with bacteria pre-treated with KYTs significantly increased larval survival compared to the untreated W83 strain (p ≤ 0.001; Fig 4A). Moreover, KYT-treated wild-type W83 were phagocytosed 2.3-fold more efficiently than untreated *Pg* in yolk (0.98% ± 0.58% and 0.43% ± 0.3% respectively; p ≤ 0.01) and 1.8-fold more efficiently in the tail region (0.97% ± 0.45% and 0.53% ± 0.3% respectively; p ≤ 0.0; Fig 4B–4E), providing compelling evidence that gingipains act to protect organisms against phagocytosis or acidification.

### Macrophages are crucial for gingipain-mediated phagocytosis of *Pg* in both systemic and local infection

**Fig 5:**
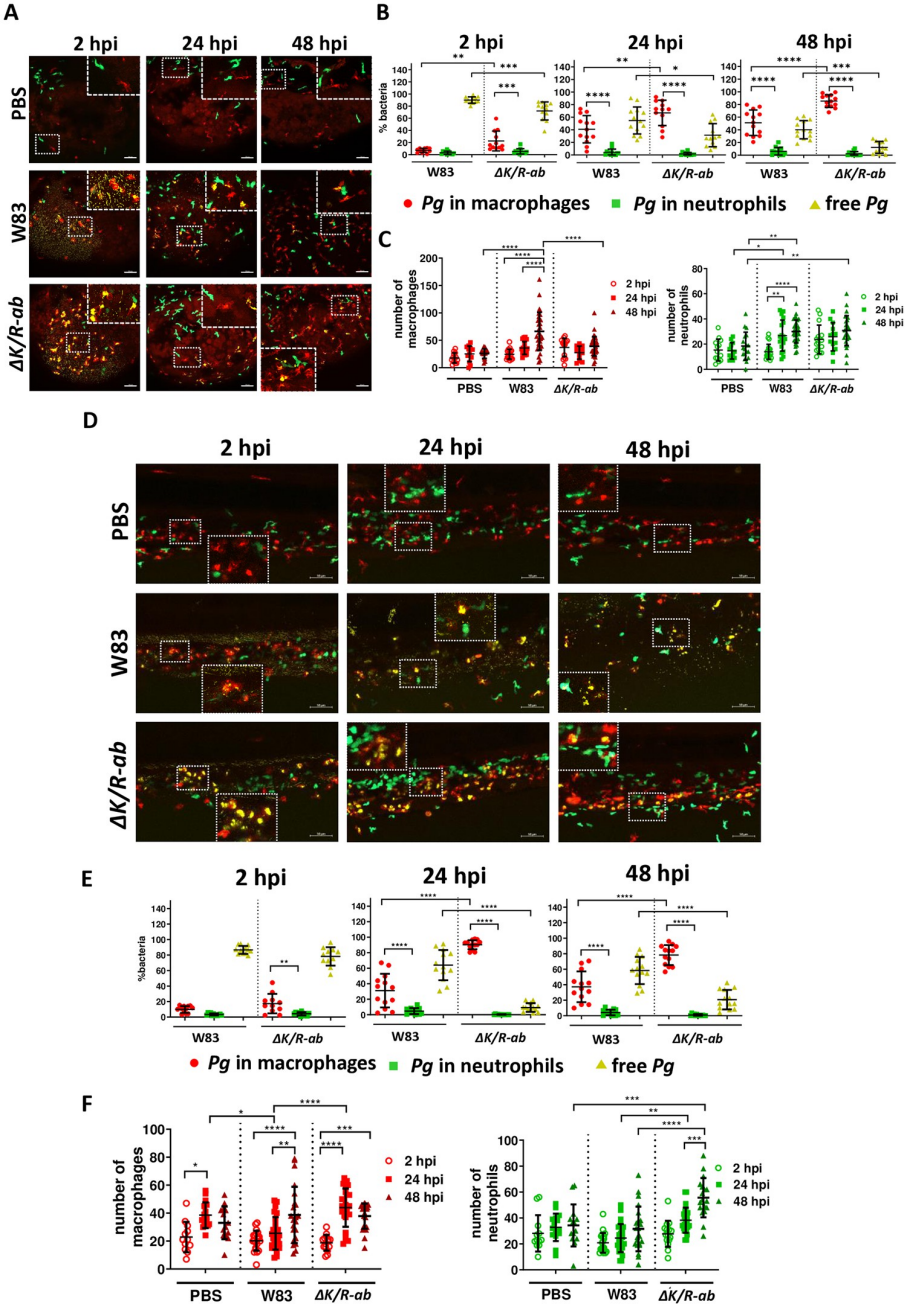
The role of macrophages and neutrophils in the eradication of *Porphyromonas gingivalis (Pg)* infection. Zebrafish larvae (30 hpf) were infected systemically with AlexaFluor647–SE labelled wild-type *Pg* W83 or *ΔK/R-ab* and real-time examination of phagocytosis was performed using transgenic zebrafish larvae *Tg(mpeg1*:*mCherry*; red macrophages) and *Tg(mpx*:*EGFP*, green neutrophils). PBS was injected as a control. Representative images of the yolk (A) or the tail (D) regions at 2, 24 or 48 hours post-infection (hpi). Scale bar = 50 μm. Quantified percentage of bacteria phagocytosed by macrophages, neutrophils and non-phagocytosed (free) bacteria at 2, 24 and 48 hpi in the yolk (B) and the tail (E) regions. Number of macrophages and neutrophils in the yolk (C) and the tail (F) regions at 2, 24 or 48 hpi. White dashed boxes correspond to zoomed regions of the images. Each dot represents quantified data from a single larva obtained in at least 3 independent experiments. Graphs show means ± SD. Differences between groups were analysed by Two-Way ANOVA.*p≤0.05,**p≤0.01,***p≤0.001,****p≤0.0001.

Having observed the importance of myeloid cells in *Pg* phagocytosis and clearance, we next aimed to further decipher the contribution of macrophages and neutrophils in *Pg* immunity by using transgenic zebrafish containing red-labelled macrophages and green-labelled neutrophils. Transgenic larvae were infected systemically with Alexa Fluor (AF)-647-SE-labelled *Pg* W83 or Δ*K/R-ab*, and real-time confocal microscopy employed to characterize the response of macrophages and neutrophils to bacterial challenge. At 2 hpi the bacteria disseminated rapidly in the larval body with only a small proportion of W83 phagocytosed by macrophages (6.9% ± 3.5%) and neutrophils (3.0% ± 2.7%) and most observed in the bloodstream as free bacteria (90% ± 5.2%). In contrast, increased phagocytosis of Δ*K/R-ab* was apparent where the gingipain-null bacteria co-localised predominantly with macrophages (Fig 5A and 5B). In comparison to wild-type W83 *Pg*, phagocytosis of Δ*K/R-ab* by macrophages, was 3-fold higher in the yolk region at 2 hpi (22.5% ± 16.3% compared to 6.9% ± 3.5%; p ≤ 0.01; Fig 5A and 5B; panel 2 hpi).

We confirmed these observations using murine RAW 264.7 macrophage and primary human monocyte-derived macrophage (hMDM) *in vitro* infection assays followed by flow cytometric and fluorescence confocal microscopy analysis. Both RAW 264.7 macrophages (S1 Fig) and hMDM (S2 Fig) displayed increased median fluorescence intensity (MFI) and therefore contained more intracellular *ΔK/R-ab* than W83. Analysis of microscopy images showed that both RAW 264.7 macrophages and hMDM infected with W83 displayed uniform phagocytosis of *Pg* with each macrophage containing only a few bacteria per cell. In contrast, macrophages phagocytosing *ΔK/R-ab* were full of bacteria (S1 and S2 Figs). These data were similar to those observed in our *in vivo* analysis of phagocytosis by zebrafish larvae.

Moreover, we observed that in zebrafish larvae at 2 hpi macrophages phagocytosed significantly more Δ*K/R-ab* than neutrophils in both yolk (p ≤ 0.001) and tail (p ≤ 0.01) regions (Fig 5A, 5B, 5D and 5E; panels 2 hpi). Similarly, at 24 hpi, in comparison to wild-type W83 *Pg*, phagocytosis of Δ*K/R-ab* by macrophages, was 1.6-fold higher in the yolk region (67.0% ± 20.1% compared to 41.0% ± 21.8%; p ≤ 0.01; Fig 5A and 5B; panel 24 hpi) and 3-fold higher in the tail region (90.4% ± 5.8% compared to 31.2% ± 21.8%; p ≤ 0.0001; Fig 5D and 5E; panel 24 hpi). Likewise, neutrophils did not significantly phagocytose neither wild-type W83 or Δ*K/R-ab Pg*. However, we observed increased number of neutrophils 24 hpi in the yolk region of *Pg* W83-infected larvae compared to 2 hpi (p ≤ 0.01; Fig 5C). In addition, we found increased numbers of neutrophils and macrophages in the tail region of Δ*K/R-ab-*infected larvae compared to the *Pg* W83-infected larvae at 24 hpi (p ≤ 0.01 and p ≤ 0.0001 for neutrophils and macrophages respectively; Fig 5F). At 48 hpi the majority Δ*K/R-ab* had been phagocytosed by macrophages (85.4% ± 10.0% in the yolk and 78.3% ± 12.9% in the tail region), compared to W83 (51.2% ± 20.1% in the yolk and 37.4% ± 19.8% in the tail; Fig 5B and 5E; panel 48 hpi). At 48 hpi, significantly more of the wild-type *Pg* was found as free bacteria (40.2% ± 14.3%), disseminated into the surrounding tissues compared to Δ*K/R-ab* bacteria (12.3% ± 9.3%; Fig 5A, 5B, 5D and 5E; panel 48 hpi).

A phagocytic response of neutrophils toward *Pg* was minimal at any time point (Fig 5B and 5E), despite their numbers within the circulation increasing as infection progresses, similar to macrophages (Fig 5C and 5F). In the case of Δ*K/R-ab* infection, we observed increased numbers of neutrophils at 48 hpi in comparison with PBS-injected controls (p ≤ 0.001) and *Pg* W83-injected larvae (p ≤ 0.0001; Fig 5D and 5F). These neutrophils were localised closely to the region of caudal hematopoietic tissue, where bacteria were phagocytosed by macrophages, suggesting emergency myelopoiesis and that these cells might play other role supporting bacterial clearance. An increased number of macrophages was also observed at this timepoint in the yolk region after *Pg* W83 but not with Δ*K/R-ab* infection (p ≤ 0.0001; Fig 5C; panel 48 hpi) but the number of neutrophils was increased in both cases in the yolk region (both p ≤ 0.01).

Phagocytosis of *Pg* W83 and Δ*K/R-ab* was further analysed by calculating the phagocytic index, reflecting the number of bacteria ingested by an individual macrophage or neutrophil (S3A and S3B Fig). The results confirmed significantly increased phagocytosis of Δ*K/R-ab* mutant compared to wild-type W83 *Pg* in the yolk at 2 hpi (p ≤ 0.0001) and 48 hpi (p ≤ 0.01) (S3A Fig), and at 24 hpi in the tail (p ≤ 0.01; S3B Fig). In addition, a significantly higher phagocytic index for Δ*K/R-ab* was observed for macrophages than neutrophils at 2, 24 and 48 hpi in the yolk (p ≤ 0.0001; S3A Fig) and at 2 and 48 hpi in the tail (p ≤ 0.05; S3B Fig). We also examined the percentage of macrophages and neutrophils actively phagocytosing *Pg*. Macrophages were more involved in *Pg* phagocytosis than neutrophils, particularly at 2 hpi (S3C and S3D Fig).

**Fig 6:**
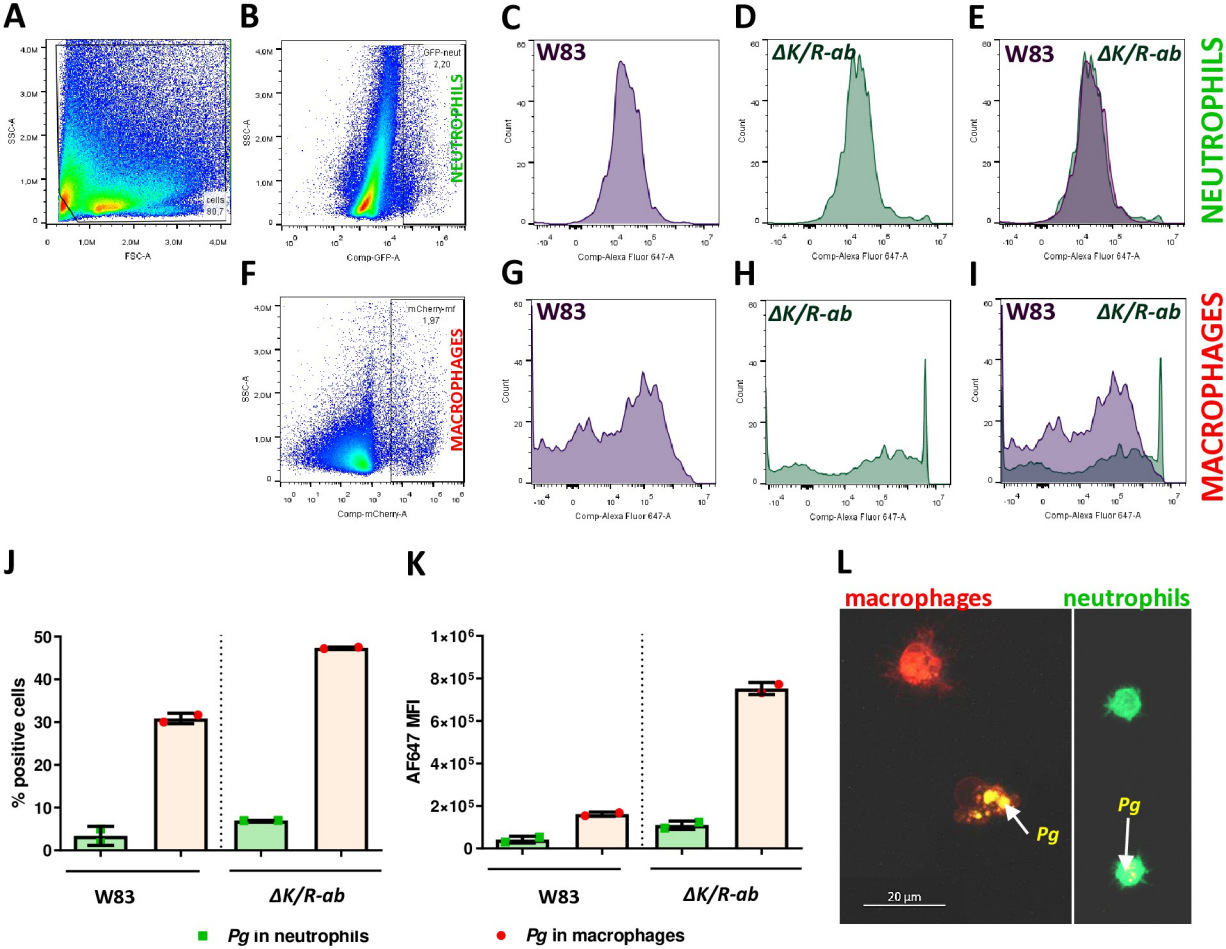
Flow cytometry analysis of phagocytosis of *Pg* W83 and *ΔK/R-ab* mutant by macrophages and neutrophils in zebrafish larvae. Transgenic zebrafish larvae *Tg(mpeg1*:*mCherry)* x *Tg(mpx*:*EGFP)* were infected with AF647-SE-stained *Pg* W83 or *ΔK/R-ab* and flow cytometry analysis was performer 24 hpi. Gating strategy used for flow cytometry analysis from zebrafish larval homogenate (A). Gating strategy used to enumerate population of EGFP+ neutrophils (B-E) or mCherry+ macrophages (F-I) 24 hpi. Percentage of neutrophils or macrophages associated with bacteria (J). Mean fluorescence intensity of neutrophils and macrophages associated with bacteria (K). Representative confocal microscopy image of cells sorted from infected larvae (L). Red–macrophages, green—neutrophils, yellow- *Pg*. hpi–hours post infection.

To validate the microscopy phagocytosis data, we took an alternative approach where *Pg* infected dual transgenic zebrafish larvae were homogenised and subjected to flow cytometric analysis (Fig 6). Similar to the microscopic analysis, we found that macrophages displayed increased phagocytosis of both W83 and *ΔK/R-ab* compared to neutrophils (~ 30% compared to 5% for W83 and 50% compared to 10% for *ΔK/R-ab*, respectively (Fig 6J). Macrophages also showed a higher median fluorescence intensity signal from engulfed bacteria than neutrophils, in particular for the *ΔK/R-ab* mutant, suggesting that these phagocytes were more proficient at phagocytosing *ΔK/R-ab* than W83 (Fig 6J and 6K). Cell sorting of macrophages and neutrophils followed by confocal microscopy analysis, reaffirmed this analysis (Fig 6L). We further analysed the role of *Pg* in phagocytosis of apoptotic neutrophils by macrophages, often referred to as efferocytosis, because gingipain-dependent defective clearance of apoptotic cells and reduced anti-inflammatory responses has been reported previously [12]. We observed very few instances of neutrophils within macrophages in zebrafish larvae in both W83 and *ΔK/R-ab*-infected fish, however, we did not observe any such events in PBS controls (S4 Fig).

**Fig 7:**
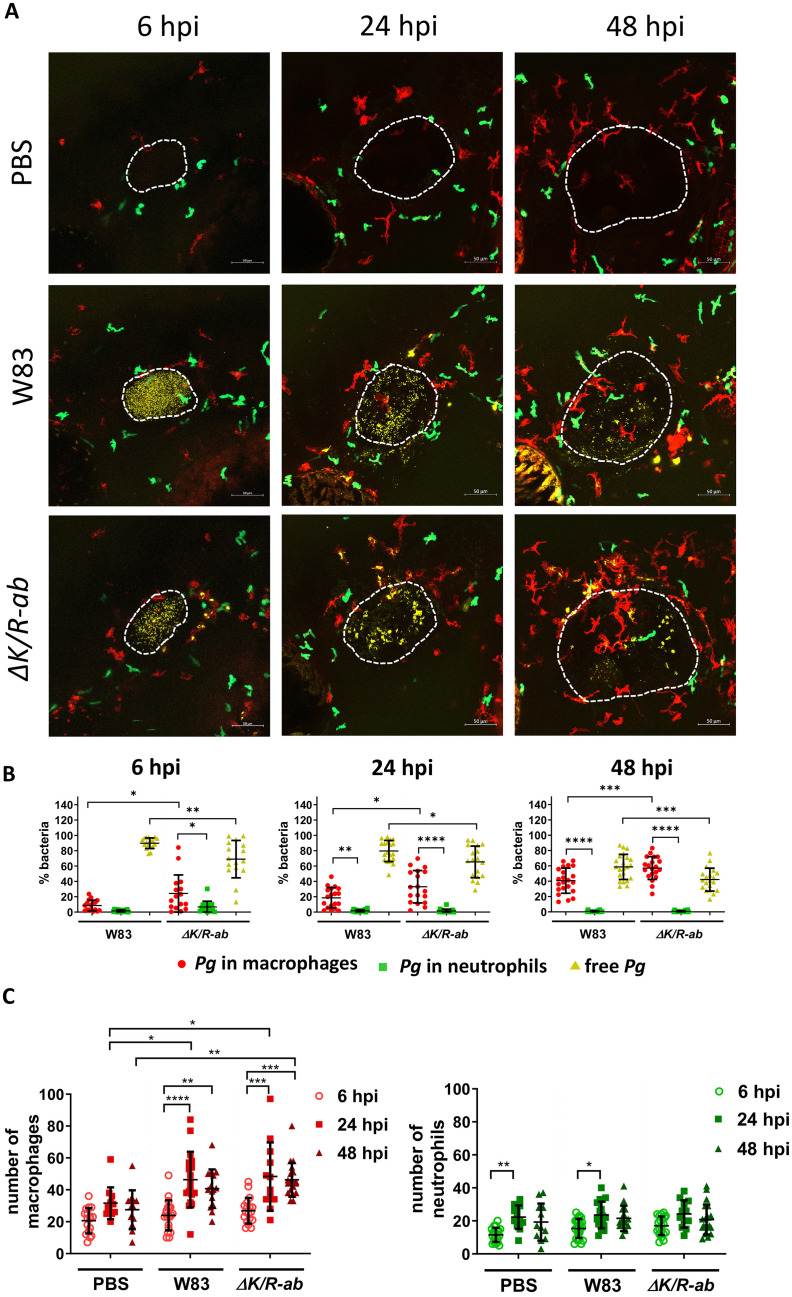
The role and migration of macrophages and neutrophils during local infection of *Porphyromonas gingivalis (Pg)* into the otic vesicle. Zebrafish larvae (30 hpf) were infected locally into the otic vesicle with AlexaFluor647–Se-labelled wild-type *Pg* W83 or *ΔK/R-ab* and real-time examination of phagocytosis and cell migration was performed using transgenic zebrafish larvae *Tg(mpeg1*:*mCherry) x Tg(mpx*:*EGFP)*. PBS was injected as a control. (A) Representative images of the otic vesicle at 6, 24 or 48 hours post-infection (hpi). Scale bar = 50 μm. White dashed lines indicate otic vesicle location. (B) Quantified percentage of bacteria phagocytosed by macrophages, neutrophils and non-phagocytosed (i.e., free *Pg*) at 2, 24 and 48 hpi in the otic vesicle and surrounding region. (C) Number of macrophages and neutrophils attracted toward the infection site. Each dot represents quantified data from a single larva obtained in at least 3 independent experiments. Graphs show means ± SD. Differences between groups were analysed by Two-Way ANOVA. *p≤0.05,**p≤0.01,***p≤0.001,****p≤0.0001.

We next evaluated if in local infection, phagocyte migration and phagocytosis of *Pg* would be different to systemic infection. Here, transgenic zebrafish larvae (30 hpf) were infected into the otic vesicle with AF647-SE-labelled *Pg* W83 or Δ*K/R-ab*. Similarly, to systemic infection, we observed that the Δ*K/R-ab* mutant was more efficiently phagocytosed than *Pg* W83 (p ≤ 0.001 by 48 hpi). Macrophages were the major phagocytic cells responsible for bacterial clearance, while neutrophils did not interact with the bacteria efficiently (Fig 7A and 7B). Compared to systemic infection, local tissue infection appeared to be cleared less effectively by 48 hpi. Moreover, there was increased migration of macrophages, but not neutrophils toward the otic vesicle in both *Pg* W83 (46.4% ± 17.4%) and Δ*K/R-ab* (48.3% ± 21.4%) infected larvae at 24 hpi compared to PBS injection (31.6% ± 10.0%; p ≤ 0.05; Fig 7C). To confirm the functionality of larval neutrophils at this stage of development and their ability to migrate toward bacteria and effectively ingest them, zebrafish were also infected with *Fusobacterium nucleatum* (*Fn*) into the otic vesicle. The ability of neutrophils to migrate toward this pathogen in zebrafish larvae was recently reported [24]. We observed 5-fold increased migration and recruitment of neutrophils to the *Fn* infection site from 2 hpi (11.3 ± 4.9 neutrophils), compared to *Pg* W83 (2.1 ± 2.3 neutrophils, p ≤ 0.0001) and PBS (control) (2.1 ± 1.3 neutrophils, p ≤ 0.0001) that remained up to 10 hpi (S5A and S5B Fig). Moreover, *Fn* and neutrophil fluorescence co-localised demonstrating effective phagocytosis of this bacterium by neutrophils (S5A Fig).

## Discussion

An association exists between periodontitis and cardiovascular to neurodegenerative diseases [18,25]. However, the mechanism of bacterial dissemination, evasion of immune control and the role of microbial virulence factors in development of these diseases remains elusive. In the case of cardiovascular disease, reports suggest that, once released into the bloodstream, *Pg* can affect the endothelium lining vascular walls. Indeed, in our previous studies, using the zebrafish systemic infection model, we showed that *Pg* and its outer membrane vesicles (OMV) can increase the vascular permeability of blood vessels via degradation of endothelial adhesion proteins in a gingipain-dependent manner [22,23,26]. In this model, *Pg* OMV also activate proinflammatory transcription pathways [27]. In order to persist in the circulation long enough to bind to the endothelium *Pg* must employ strategies to resist engulfment from circulating phagocytes or to avoid destruction by acidified phagolysosomes once internalised. Therefore, determining how *Pg* interact with phagocytes is important.

Using a zebrafish larvae systemic infection model coupled with real-time imaging and bacterial recovery assay, we found that the well characterised isogenic *Pg* mutant *ΔK/R-ab* that is devoid of gingipains [26,28] were eradicated much faster by zebrafish larvae than wild-type W83 bacteria with intact gingipains. It should be noted that previous studies confirmed that there are no phenotypic changes between *ΔK/R-ab* and its parent strain, W83, except for gingipain activity [22,29–31] discounting this as a factor. Moreover, we showed that infection with *ΔK/R-ab* did not cause larval zebrafish mortality and development of systemic disease, in contrast to the highly pathogenic wild-type bacteria, implicating gingipains as a key virulence factor in the disease process [22]. The observation of the rapid eradication of the *ΔK/R-ab* may further explain this finding. In support, many studies indicate that gingipains play a crucial role in *Pg* survival. These proteases have been shown to take part in processes such as deriving substrates for bacterial growth, overcoming oxidative stress and host immune response [32], protecting the bacterium from unfavourable environments. Yamatake *et al*. showed that wild-type *Pg* is much more persistent in human artery endothelial cells, compared to a gingipain-null mutant KDP136, and observed that this mutant was more susceptible to killing within phagolysosomes of innate immune cells than its wild-type counterpart [33].

It is well documented that phagocytes such as macrophages and neutrophils play a key role in the innate immune response to *Pg* at periodontal sites and in systemic murine models of *Pg* infection [5–8,34]. In this study, ablation of myeloid cells in *pu*.*1* morpholino zebrafish caused increased susceptibility to both wild-type *Pg* and *ΔK/R-*ab infection compared to control larvae. Moreover, the rapid mortality of phagocyte-depleted embryos infected with wild-type *Pg*, and to a lesser extend with *ΔK/R-*ab, highlight the importance of phagocytes in clearing of *Pg*, and also gingipains in pathogenesis. Phagocytes are not only responsible for eradicating pathogens but also produce potent proinflammatory mediators that drive immune responses–but when this inflammation is dysregulated, they are largely responsible for causing tissue damage. To the latter end, macrophage-depleted mice show decreased bone resorption and a decreased level of proinflammatory cytokines after oral infection with *Pg* [34].

pHrodo-Red dye, was used to determine the specific phagosomal location of *Pg*. This allows bacteria destined to be killed in acidified phagosomes (phagolysosomes) to be distinguished from bacteria in other intracellular cell locations that may survive internalisation as well as non-internalised bacteria. We found the increased presence of *ΔK/R-ab* and wild-type *Pg* treated with the highly specific gingipain inhibitors KYT-1 and KYT-36 [10,35,36] in acidified phagosomes, in comparison to the wild-type bacteria, indicating the key role of the proteolytic activity of gingipains in preventing bacterial acidification. The low numbers of wild-type *Pg* in acidified phagosomes may be caused by decreased bacterial phagocytic uptake due to affected binding of the bacterium at the cell surface by pathogen recognition receptors (PRR), disruption of PRR-associated molecules or altered transport of bacteria to phagosomes once internalised. In support of this, we observed increased phagocytosis of *ΔK/R-ab* mutant by macrophages compared to the wild-type *Pg* W83 in zebrafish *in vivo* by quantitative microscopy and flow cytometry and by murine RAW 264.7 macrophages and hMDM *in vitro*.

The specific role of gingipains in inhibiting phagocytosis is still unclear although several potential mechanisms might explain this observation. Wild-type *Pg* has been shown to degrade opsonins and host cell surface proteins including receptors on phagocytic cells, that may prevent host-bacteria interaction, intracellular signalling and thus phagocytosis [37,38]. For example, work by Castro *et al*. and Wilensky *et al*. showed gingipain-mediated cleavage of CD14 [12,37], a key PRR co-receptor that, along with TLR2, not only drives intracellular signalling leading to NFκB activation [12] but also mediates phagocytosis of Gram-negative bacteria [39–41]. Reports show that *Pg* can also modulate phagocytosis by capsule-dependent evasion [42], and/or suppression of phagolysosome maturation via the TLR2-PI3K pathway [43]. Our data also complement the finding of Sundqvist *et al*., who showed that W83 amongst other *Pg* stains is resistant to phagocytosis [44]. In addition, Yamatake *et al*., found slower maturation of phagosomes that contained wild-type *Pg*, in contrast to phagosomes containing a gingipain-null mutant [33]. It is therefore plausible that *Pg* cleaves macrophage receptors and other surface proteins, protecting *Pg* from phagocytosis and eradication via acidified phagosomes in a gingipain-dependent manner.

Our results using transgenic zebrafish with fluorescently labelled phagocytes indicate that macrophages play a predominant role in phagocytosis of *Pg* in zebrafish larvae. In humans, periodontal-associated bacteria interact with macrophages contributing to both inflammation and tissue homeostasis. Depending on the environmental cues, macrophages display incredible plasticity and can adjust their phenotype accordingly, functioning as master regulators of periodontal tissue homeostasis [45]. Importantly, increased levels of monocytes in the blood correlates with better outcomes in sepsis patients indicating that these phagocytes play a crucial role in clearing systemic infection [46]. We observed macrophages full of engulfed *Pg* circulating within the bloodstream, where they have the potential to migrate to distant tissues. *In vitro* studies suggest that *Pg* can survive and escape from inflammation resolving M2 macrophages but not pro-inflammatory M1 cells, which suggests that inflammation resolving but not inflammatory macrophages can act as Trojan horses in bacterial dissemination [21]. However, further studies are warranted to evaluate if this mechanism occurs within zebrafish macrophages and therefore contribute to bacterial dissemination.

Using the systemic and otic infection models we observed fast and efficient phagocytosis of *Pg* by macrophages with only minimal phagocytosis by neutrophils despite presence of gingipains. *In vitro* studies show that *Pg* can subvert neutrophil functions such as degradation of chemotactic factors that drive their migration or inefficient phagocytosis [47,48]. *In vivo* we similarly showed that neutrophils are not efficiently recruited toward *Pg* and do not effectively phagocytose these bacteria during systemic or local tissue infection. It is unlikely that this is due to lack of neutrophil maturity since the same neutrophils were recruited toward and phagocytosed the oral pathobiont *Fusobacterium nucleatum*, a finding also observed by Ellet *et al*., [24]. In addition, although efferocytosis was out of the scope of this manuscript, our current data may indicate that such process may exist upon *Pg* infection in zebrafish and is worthy of further investigation.

Neutrophils are known to accumulate in large numbers at sites of periodontitis in humans, where they play an important role in combating bacterial infection [7]. This may seem contradictory to the data presented here where the pathobiont, *Pg*, does not appear to induce neutrophil recruitment and these cells do not phagocytose *Pg* in large numbers in contrast to macrophages, despite an overall increase in neutrophil numbers, which may indicate more subtle but complex function of neutrophils to be further investigated. However, it must be noted that periodontitis is a polymicrobial disease where *Pg* is one of many pathobionts within the disease-causing biofilm. Indeed, although *Pg* is known as a ‘keystone pathogen’ its presence within periodontal biofilm is low compared to other inflammation-promoting bacteria, and thus the effects of gingipains in this multispecies environment may be limited. However, the biofilm disaggregates and disperses to generate either small bacterial aggregates or single species that then enter the circulation. It is here, in these small colonies or as individual bacteria, that the actions of *Pg* and their gingipains may come to the fore and be more prominent in causing systemic or neurodegenerative disease. In finding that macrophages out-weigh neutrophil function, our study provides new insights into the host innate immune response toward *Pg* and identify gingipains as potential targets for therapeutic intervention for periodontal-mediated cardiovascular or neurodegenerative disease.

Collectively, our results highlight the interplay and likely competition between macrophages and neutrophils in combating systemic and tissue *Pg* infection and uncover the role of gingipains in subverting the innate immune response. With the understanding that phagocytes in experimental animal models may not directly replicate those of humans *in vivo*, our study demonstrates the advantages of using zebrafish to study phagocyte-pathogen interactions *in vivo*, providing valuable information that may lead to the further understanding of the pathogenic mechanisms and potential therapeutic strategies in periodontal-mediated diseases.

## Materials & methods

### Ethics statement

All experiments were conducted in accordance with the European Community Council Directive 2010/63/EU for the Care and Use of Laboratory Animals of Sept. 22, 2010 (Chapter 1, Article 1 no.3) and National Journal of Law act of Jan. 15, 2015, for Protection of animals used for scientific or educational purposes (Chapter 1, Article 2 no.1). All methods involving zebrafish embryos/larvae were following ARRIVE guidelines. Zebrafish maintenance and experimental work was performed in accordance with UK Home Office regulations and UK Animals (Scientific Procedures) Act 1986. The Jagiellonian University Zebrafish Core Facility (ZCF) is a licensed breeding and research facility (District Veterinary Inspectorate in Krakow registry; Ministry of Science and Higher Education record no. 022 and 0057).

### *Pg* strains and culture conditions

Wild-type *Pg* W83 strain, its isogenic gingipain mutants *(ΔK/R-ab*, *kgp*^Δ598–1732^::Tc^r^ *rgpA*^-^::Cm^r^ *rgpB*^Δ410–507^::Em^r^) [31] and *Fusobacterium nucleatum* ATTC 10953 were maintained in Triptic Soy Broth (TSB; Sigma-Aldrich, USA) anaerobic blood agar (ABA) plates supplemented with 0.5 mg/L menadione sodium bisulphate, 0.25 g/L L-cysteine-HCl (BioShop, Canada), 5 mg/L hemin (Sigma-Aldrich, USA), 5% (v/v) defibrinated sheep blood (Oxoid, Basingstoke, UK) and antibiotic (1 μg/mL tetracycline and 5 μg/mL erythromycin) when appropriate. For liquid cultures, these bacteria were grown in hemin, menadione and L-cysteine supplemented TSB broth. Bacteria were cultured at 37°C in an anaerobic environment (80% N_2_, 10% CO_2_ and 10% H_2_) and re-plated every 4–5 days. Prior to larvae microinjection, *Pg* or *Fn* were grown in liquid cultures overnight at 37°C in anaerobic conditions. For *Pg* fresh cultures were set to an optical density (OD_600_) equal to 0.1 and cultured until log phase. *Pg* and *Fn* were harvested by centrifugation at 6,000 × g for 10 minutes, washed and re-suspended in PBS, pH 7.4 at OD_600_ 5.0.

### *Pg* treatment with KYT inhibitors of gingipains

*Pg* were pre-treated with the specific gingipain inhibitors KYT-1 and KYT-36 (Peptide Institute, Japan) [10,36] at a final concentration of 2 μM and were incubated for 30 minutes at 37°C in anaerobic conditions. Bacteria were washed with PBS and resuspended at OD_600_ = 5.0 to inject into zebrafish larvae. Loss of gingipain activity was confirmed using a gingipain activity test as described previously [49].

### Zebrafish husbandry, lines, and experimental conditions

Adult zebrafish were kept at 28°C in a continuous re-circulating closed aquarium system in 14:10 hours light/dark cycle. Adults were fed with live artemia (Artemia Koral, Germany) or commercial feed (Gemma Micro 300ZF, Skretting, Stavanger, Norway) twice daily. Unless otherwise stated, the London Wild-Type (LWT) zebrafish obtained from The Bateson Centre, University of Sheffield, were used for all experiments. Larvae were maintained in E3 medium at 31°C, according to standard protocols and monitored for up to 4 days post-fertilization (dpf). Studies using transgenic *Tg* (*mpeg1*:*mCherry)ump* [50] and *Tg (mpx*:*EGFP)* [51] were performed at the Institute of Zoology and Biomedical Research, Jagiellonian University Zebrafish Core Facility.

### Systemic and local microinjection of *Pg* into zebrafish larvae

Non-filament borosilicate microcapillary glass injection needles were pulled to obtain a well-defined injection tip. The needle was filled with 5–10 μL of bacterial suspension at a known concentration and zebrafish larvae at 30 hours post-fertilisation (hpf) were microinjected systemically (Pneumatic PicoPump microinjector, World Precision Instruments, USA) with 3 nL of *Pg* into the common cardinal vein (Duct of Cuvier); PBS-injected larvae were used as control [22]. To induce local bacterial infection, 0.5 nL of *Pg* suspension was injected to the otic vesicle of zebrafish larvae. To confirm doses of *Pg* infected, bacteria were injected into PBS and viable counts determined on ABA plates after growth in anaerobic conditions.

### Determination of *Pg* persistence in zebrafish larvae

*Pg* were labelled with 10 mg/mL fluorescein-5-EX N-hydroxysuccinimide ester (fluorescein-SE, ThermoFisher Scientific, USA) then injected systemically as described previously, followed observation using Light-Sheet Microscopy (Zeiss Z1 Light Sheet Microscope with ZenBlack 2014 software, Carl Zeiss, Germany) with Z-stack time-series acquired every 10 minutes for 12 hours. Video images were created using ImageJ (National Institute of Health, USA) and maximum intensity Z-stacks of selected time-points analysed. Fluorescein-SE-stained *Pg* infection larvae were visualized using a 488–30 laser with laser blocking filter 405/488/561. To evaluate *Pg* viability, zebrafish larvae microinjected with wild-type or *ΔK/R-ab* mutant, were individually transferred to 100 μL E3 medium in microtubes, frozen at -80°C at indicated time-points. Larvae were then homogenized, serially diluted in PBS, plated on ABA blood agar plates supplemented with 25 μg/ml gentamicin (ThermoFisher Scientific, USA) to inhibit contamination with other bacteria and incubated for 5–7 days in anaerobic conditions until visible colonies appeared, which were then enumerated.

### Determination of *Pg* acidification using a pH-sensitive dye

To monitor bacterial phagocytosis, *Pg* were dual labelled with fluorescein-SE and pHrodo red succinimidyl ester (ThermoFisher Scientific, USA). 200 μL bacterial suspension of *Pg* (in PBS; pH 9) was simultaneously mixed with 0.25 μL fluorescein-SE (10 mg/mL) and 0.25 μL of pHrodo red (1.7 mg/mL) for 30 minutes at 37°C in the dark with shaking. pHrodo is non-fluorescent until it is acidified within phagosomes where upon it fluoresces red. Bacteria were washed twice with PBS (pH 8) to remove excess dye, resuspended in PBS (pH 7.4) to an OD_600_ 5.0 and 3 nL of the suspension was microinjected into zebrafish larva. At 2 or 24 hpi, larvae were anesthetized, mounted in 0.8% low-melting-point agarose (Sigma-Aldrich, USA) in a glass capillary and visualized with a Zeiss Z1 Light Sheet Microscope. Fluorescein-SE stained *Pg* were excited using 488 laser and pHrodo red bacteria with 561 laser and blocking filter 405/488/561. To quantify *Pg* acidification, an area of interest was drawn in the yolk and tail region of maximum intensity projection images. pHrodo signal was thresholded and total area was also measured. Data was presented as a pHrodo signal/selected area. Additionally, the size of pHrodo-positive particles was also measured. To evaluate the signal from nonacidified bacteria, the pHrodo signal was subtracted from the total fluorescein-SE positive bacteria and total area was measured.

### Morpholino injection into zebrafish embryos to deplete phagocytic cells

Morpholino oligonucleotides (MO) can be used to transiently silence target genes in zebrafish. A translational blocker MO for *pu*.*1* transcriptional activator gene was used to deplete larvae of myeloid cell lineage. The *pu*.*1* knockdown experiments were performed as described previously [52]. Pu.1 MO at a volume of 0.5 nL and concentration of 1 mM was microinjected into the yolk sac of 1–2 cell stage zebrafish embryos. After injection embryos were collected, incubated in E3 with methylene blue, and any abnormal or dead larvae were discarded. Morphants were injected with *Pg* at 30 hpf as described previously.

### *Pg* interaction with macrophages and neutrophils

To examine *Pg* interaction with macrophages and neutrophils, transgenic zebrafish *Tg* (*mpeg1*:*mCherry)* and *Tg (mpx*:*EGFP)* were used and the interaction visualised by a Zeiss LSM 900 confocal microscope with an Airyscan 2 detector and a 40x NA 1.2 plan-apochromatic water immersion objective. Observations were made at 2-, 24- and 48-hpi. Zebrafish larvae were anesthetized and positioned laterally a on glass-bottomed dish in 1% low-melting point agarose in E3 medium. Maximum intensity Z-stacks were presented as representative images. The number of macrophages or neutrophils and percent of bacteria within macrophages and neutrophils or non-phagocytosed free bacteria were analysed using ImageJ. To evaluate the percentage of *Pg* phagocytosed by macrophages, neutrophils or free bacteria, the colocation of Alexa Fluor-647 NHS Ester (ThermoFisher Scientific, USA, final concentration of staining 17 μg/mL) positive signal (bacteria) with mCherry (macrophages) or GFP (neutrophils) was measured. The fluorescent signal of bacteria that did not colocalise with macrophages or neutrophils was considered as “free bacteria”. Manual counting was employed to enumerate the number of macrophages and neutrophils in the analysed images. Phagocytosis rate was expressed using a phagocytic index calculated as the percentage of macrophages or neutrophils engulfing *Pg* multiplied by the mean area of fluorescence of engulfed bacteria (i.e., total bacterial fluorescent area × percentage of phagocytic macrophages or neutrophils) based on [53]. For flow cytometric analysis, approximately 250 transgenic *Tg*(*mpeg1*:*mCherry)* x *Tg (mpx*:*EGFP)* zebrafish larvae were infected with Alexa Fluor-SE-stained *Pg* W83 or *ΔK/R-ab* as described previously. At 24 hpi, larvae were anesthetised, homogenised and subjected to flow cytometric cell sorting and analysis (Cyteck Aurora CS) as described in [54].

### Statistical analysis

All analysis was performed using Graphpad Prism v9.5.1 (GraphPad Software Inc., USA). Data are presented as the mean ± SD of three individual experiments, unless otherwise stated. Survival data were evaluated using the Kaplan-Meier method and comparisons between individual curves were made using the log rank test. Differences between two groups were measured using two-tailed, unpaired t-test with Welch’s correction or Mann-Whitney test when data did not fall into normal distribution. Multiple groups were compared using parametric One-way ANOVA or Kruskal-Wallis ANOVA when data not normally distributed. Multiple parameters in multiple groups were analysed by Two-way ANOVA. Statistical significance was assumed if p ≤ 0.05.

## Supporting information

S1 Fig Phagocytosis of *Pg* W83 and *ΔK/R-ab* by RAW 264.7 murine macrophages. Raw cells were infected in serum free medium with AF647-stained *Pg* W83 or *ΔK/R-ab* at MOI of 1:100 for indicated time and subjected to flow cytometry analysis. Gating strategy used for flow cytometry analysis to distinguish live population of RAW cells (A). Gating strategy used to analyse cells, which phagocyted bacteria 60 minutes post-infection (AF647 positive) (B-D). Differences in fluorescence intensity showed by population of RAW cells which phagocytosed *Pg* W83 (violet) or *ΔK/R-ab* (green) 60 min post-infection (E). Median fluorescence intensity of RAW cells associated with bacteria (F). Representative confocal microscopy images of RAW cells infected with *Pg* W83 or *ΔK/R-ab* (yellow) 12 h post-infection showing differences in cell morphology (red line) (G).
(TIF)

S2 Fig Phagocytosis of *Pg* W83 and *ΔK/R-ab* by human monocyte-derived macrophages (hMDM). hMDM cells were infected in serum free medium with AF647-stained *Pg* W83 or *ΔK/R-ab* at MOI of 1:100 for indicated time and subjected to flow cytometry analysis. Gating strategy used for flow cytometry analysis to distinguish population of hMDM (A). Gating strategy used to analyse cells, which phagocyted bacteria 60 minutes post-infection (AF647 positive) (B-D). Differences in fluorescence intensity showed by population of macrophages which phagocytosed *Pg* W83 (violet) or *ΔK/R-ab* (green) 60 min post-infection (E). Median fluorescence intensity of macrophages associated with bacteria (F). Representative confocal microscopy images of hMDM infected with *Pg* W83 or *ΔK/R-ab* (yellow) 12 h post-infection showing differences in cell morphology (red line) (G).
(TIF)

S3 Fig The role of macrophages and neutrophils in the clearance of *Pg* upon systemic infection. Zebrafish larvae were infected systemically with AlexaFluor647–SE labelled wild-type *Pg* W83 or *ΔK/R-ab at* 30 hpi and real-time examination of phagocytosis was performed using transgenic zebrafish larvae *Tg(mpeg1*:*mCherry*; red macrophages) and *Tg(mpx*:*EGFP*, green neutrophils). Phagocytic index in macrophages or neutrophils at 2, 24 and 48 hpi in the yolk (A) and the tail (B) regions. Percentage of macrophages and neutrophils phagocytosing *Pg* at 2, 24 and 48 hpi in the yolk (C) and the tail (D) regions. Each dot represents quantified data from a single larva obtained in at least 3 independent experiments. Graphs show means ± SD. Differences between groups were analysed by Two-Way ANOVA (A&B) or Mann-Whitney test (C and D). *p≤0.05, **p≤0.01, p≤0.001.
(TIF)

S4 Fig The effect of systemic *Pg* infection on efferocytosis. Zebrafish larvae were infected systemically with AlexaFluor647–SE labelled wild-type *Pg* W83 or *ΔK/R-ab at* 30 hpi and real-time imaging was performed using transgenic zebrafish larvae *Tg(mpeg1*:*mCherry)*; red macrophages and *Tg(mpx*:*EGFP)*; green neutrophils. PBS was injected as a control. Representative confocal microscopy images showing potential efferocytosis (white arrows) in the yolk region at 48 hpi (A). Percentage of larvae showing events of efferocytosis in the analysed region (tail or yolk) at 2, 24 and 48 hpi (B). n = 9–16 larvae. Scale bar = 20 μm. hpi-hours post infection.
(TIF)

S5 Fig The neutrophil migration during local infection of *Porphyromonas gingivalis (Pg)* and *Fusobacterium nucleatum (Fn)* into the otic vesicle. Zebrafish larvae (30 hpf) were infected locally into the otic vesicle with Alexa Fluor 647–SE labelled *Pg* or *Fn* and real-time examination of neutrophil migration was performed using transgenic zebrafish larvae *Tg(mpx*:*EGFP)*. PBS was injected as a control. (A) Representative images of the otic vesicle at 2, 6 or 10 hpi. Scale bar = 20 μm. White dashed lines indicate otic vesicle location. (B) Quantified number of neutrophils attracted toward the infection side at 2, 6 and 10 hpi. Each dot represents quantified data from a single larva obtained in 2 independent experiments. Graphs show means ± SD. Differences between groups were analysed by Two-Way Anova. *p ≤ 0.05, **p ≤ 0.01, *** p ≤ 0.001, ****p ≤ 0.0001. hpi-hours post infection.
(TIF)
