# Supplementary figures and images for: Gingipains protect Porphyromonas gingivalis from macrophage-mediated phagocytic clearance
Source: PLoS Pathog. 2025 Jan 21;21(1):e1012821. doi: 10.1371/journal.ppat.1012821 (PMC11801703; doi:10.1371/journal.ppat.1012821)

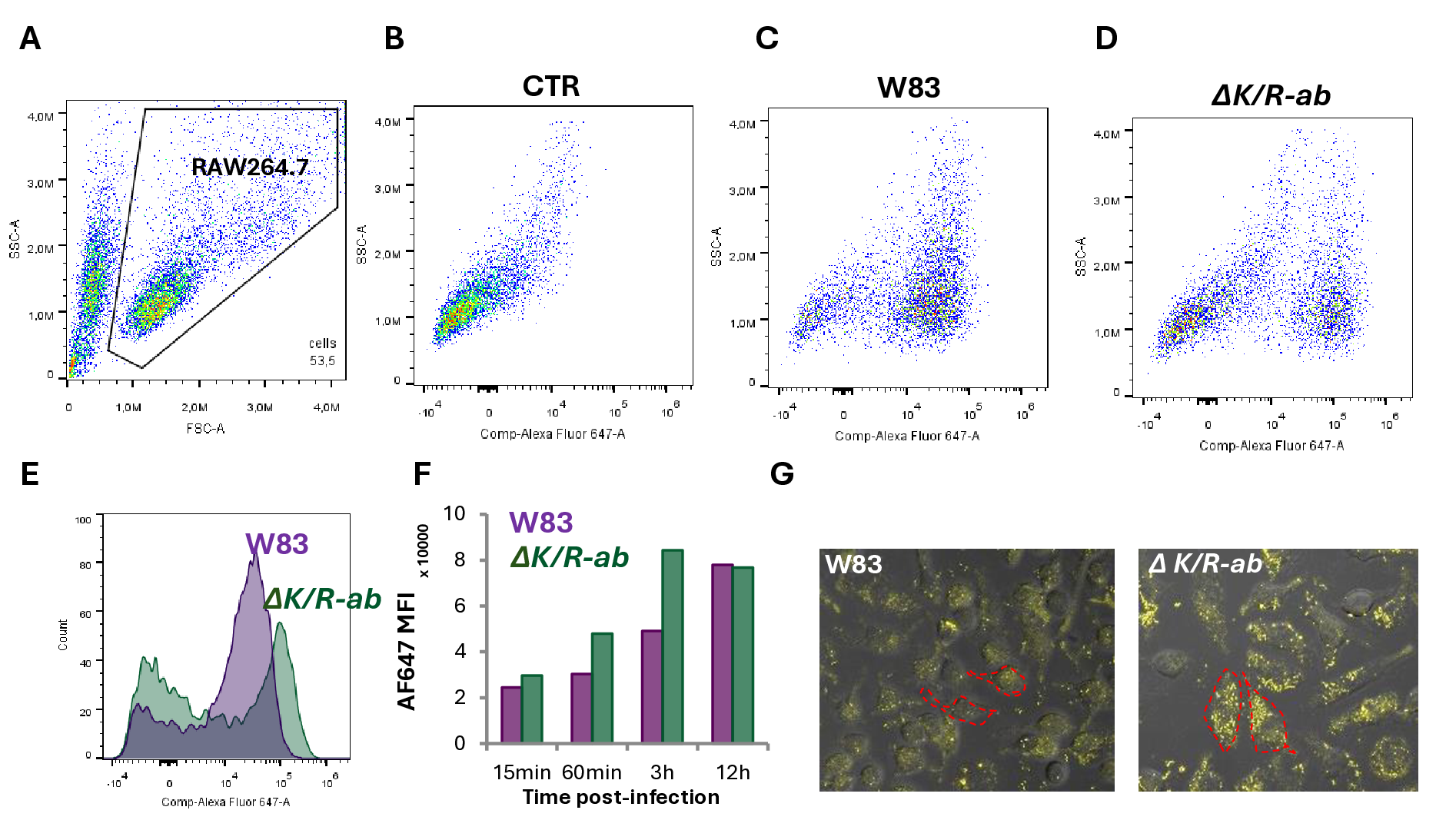

Supplement: S1 Fig — Raw cells were infected in serum free medium with AF647-stained Pg W83 or ΔK/R-ab at MOI of 1:100 for indicated time and subjected to flow cytometry analysis. Gating strategy used for flow cytometry analysis to distinguish live population of RAW cells (A). Gating strategy used to analyse cells, which phagocyted bacteria 60 minutes post-infection (AF647 positive) (B-D). Differences in fluorescence intensity showed by population of RAW cells which phagocytosed Pg W83 (violet) or ΔK/R-ab (green) 60 min post-infection (E). Median fluorescence intensity of RAW cells associated with bacteria (F). Representative confocal microscopy images of RAW cells infected with Pg W83 or ΔK/R-ab (yellow) 12 h post-infection showing differences in cell morphology (red line) (G). (TIF) [file ppat.1012821.s001.tif]

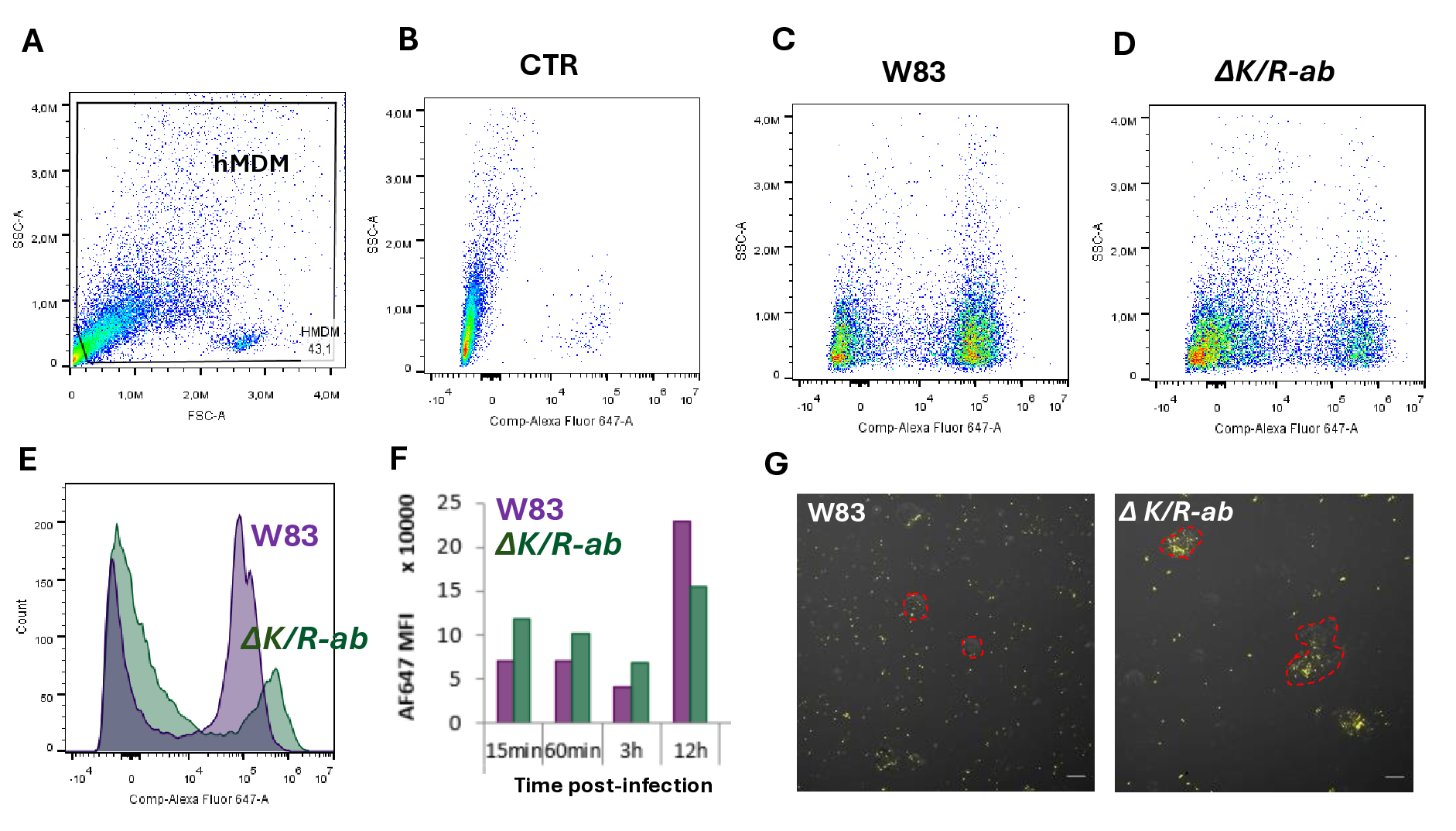

Supplement: S2 Fig — hMDM cells were infected in serum free medium with AF647-stained Pg W83 or ΔK/R-ab at MOI of 1:100 for indicated time and subjected to flow cytometry analysis. Gating strategy used for flow cytometry analysis to distinguish population of hMDM (A). Gating strategy used to analyse cells, which phagocyted bacteria 60 minutes post-infection (AF647 positive) (B-D). Differences in fluorescence intensity showed by population of macrophages which phagocytosed Pg W83 (violet) or ΔK/R-ab (green) 60 min post-infection (E). Median fluorescence intensity of macrophages associated with bacteria (F). Representative confocal microscopy images of hMDM infected with Pg W83 or ΔK/R-ab (yellow) 12 h post-infection showing differences in cell morphology (red line) (G). (TIF) [file ppat.1012821.s002.tif]

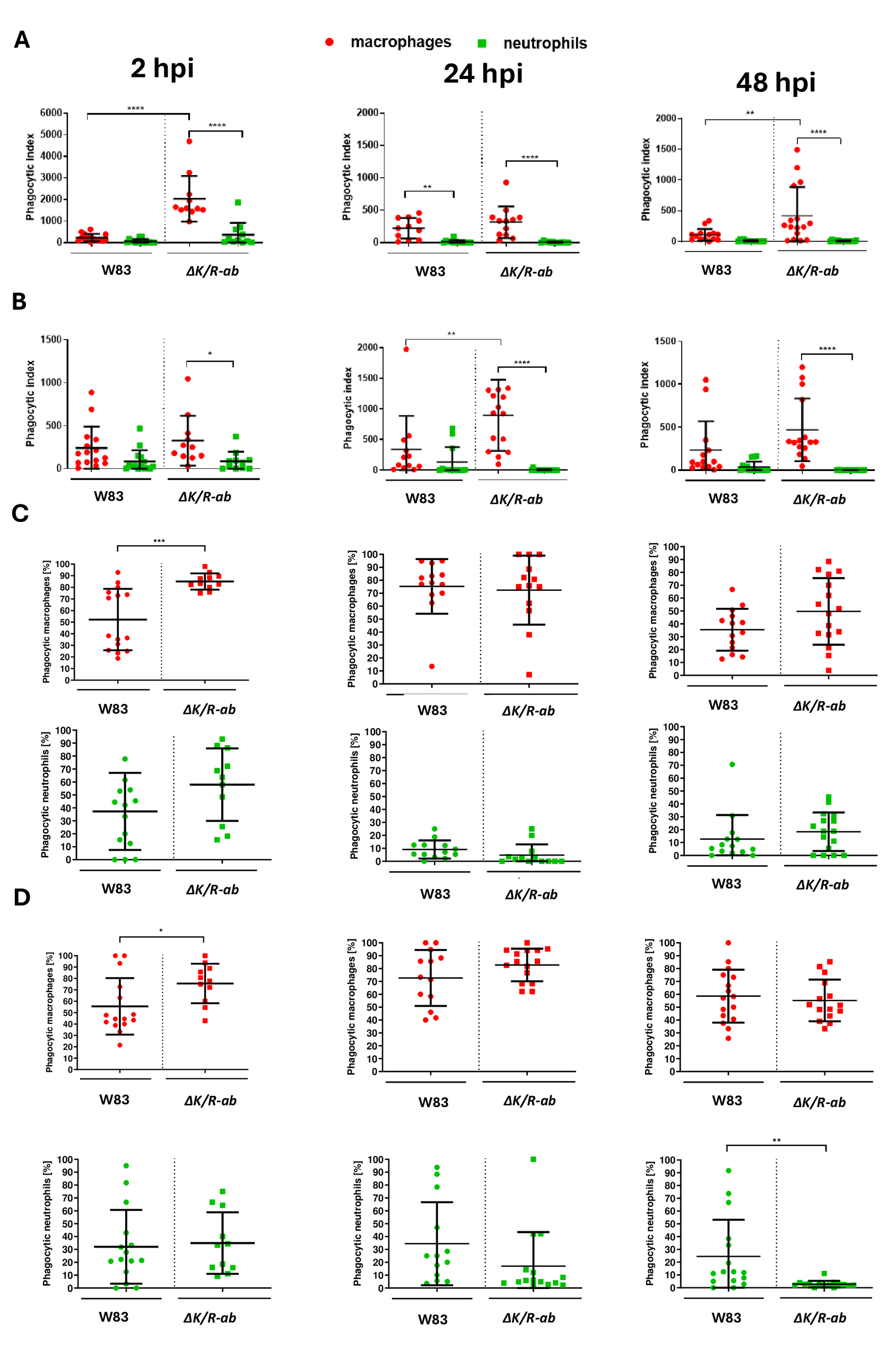

Supplement: S3 Fig — Zebrafish larvae were infected systemically with AlexaFluor647–SE labelled wild-type Pg W83 or ΔK/R-ab at 30 hpi and real-time examination of phagocytosis was performed using transgenic zebrafish larvae Tg(mpeg1:mCherry; red macrophages) and Tg(mpx:EGFP, green neutrophils). Phagocytic index in macrophages or neutrophils at 2, 24 and 48 hpi in the yolk (A) and the tail (B) regions. Percentage of macrophages and neutrophils phagocytosing Pg at 2, 24 and 48 hpi in the yolk (C) and the tail (D) regions. Each dot represents quantified data from a single larva obtained in at least 3 independent experiments. Graphs show means ± SD. Differences between groups were analysed by Two-Way ANOVA (A&B) or Mann-Whitney test (C and D). *p≤0.05, **p≤0.01, p≤0.001. (TIF) [file ppat.1012821.s003.tif]

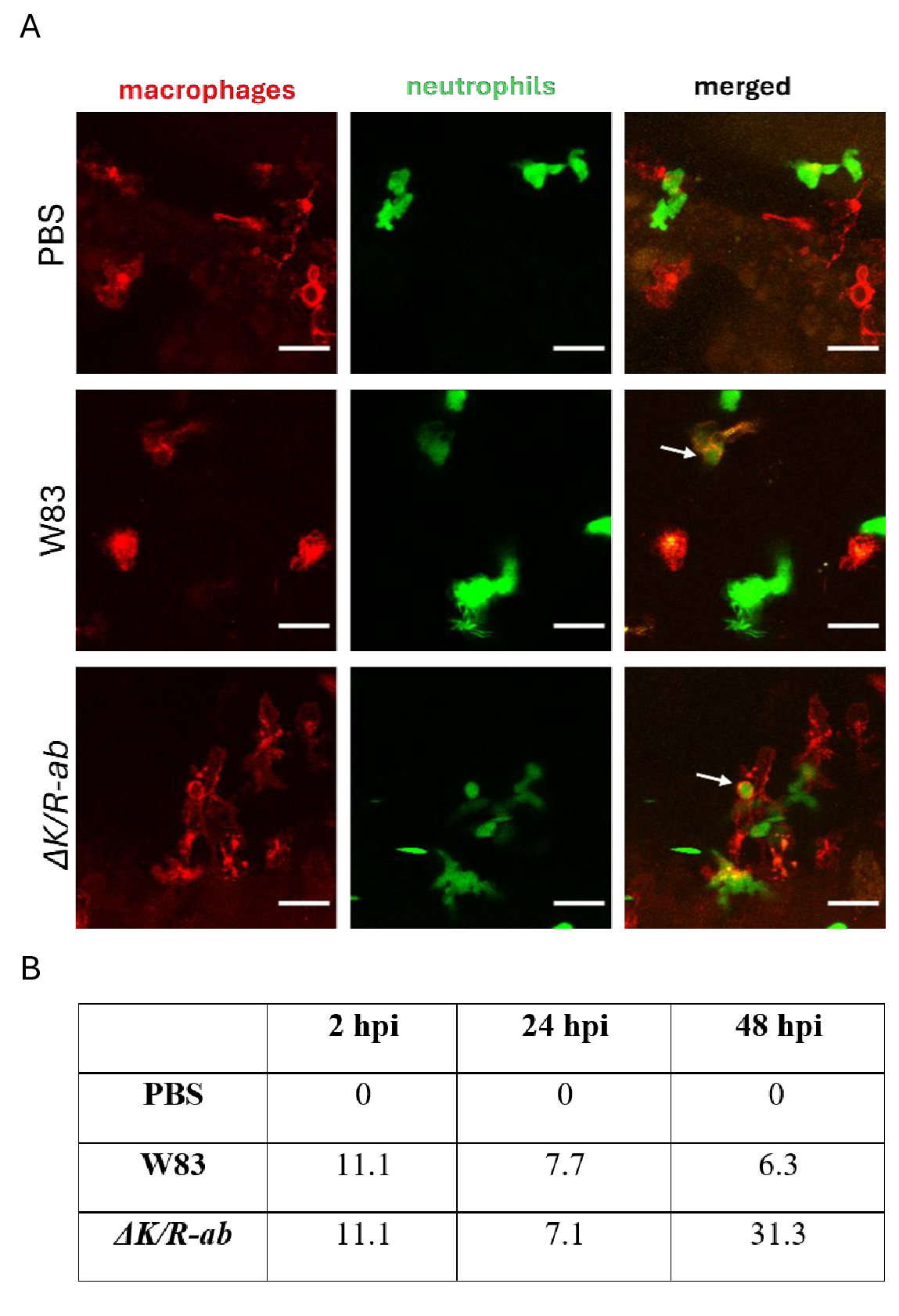

Supplement: S4 Fig — Zebrafish larvae were infected systemically with AlexaFluor647–SE labelled wild-type Pg W83 or ΔK/R-ab at 30 hpi and real-time imaging was performed using transgenic zebrafish larvae Tg(mpeg1:mCherry); red macrophages and Tg(mpx:EGFP); green neutrophils. PBS was injected as a control. Representative confocal microscopy images showing potential efferocytosis (white arrows) in the yolk region at 48 hpi (A). Percentage of larvae showing events of efferocytosis in the analysed region (tail or yolk) at 2, 24 and 48 hpi (B). n = 9–16 larvae. Scale bar = 20 μm. hpi-hours post infection. (TIF) [file ppat.1012821.s004.tif]

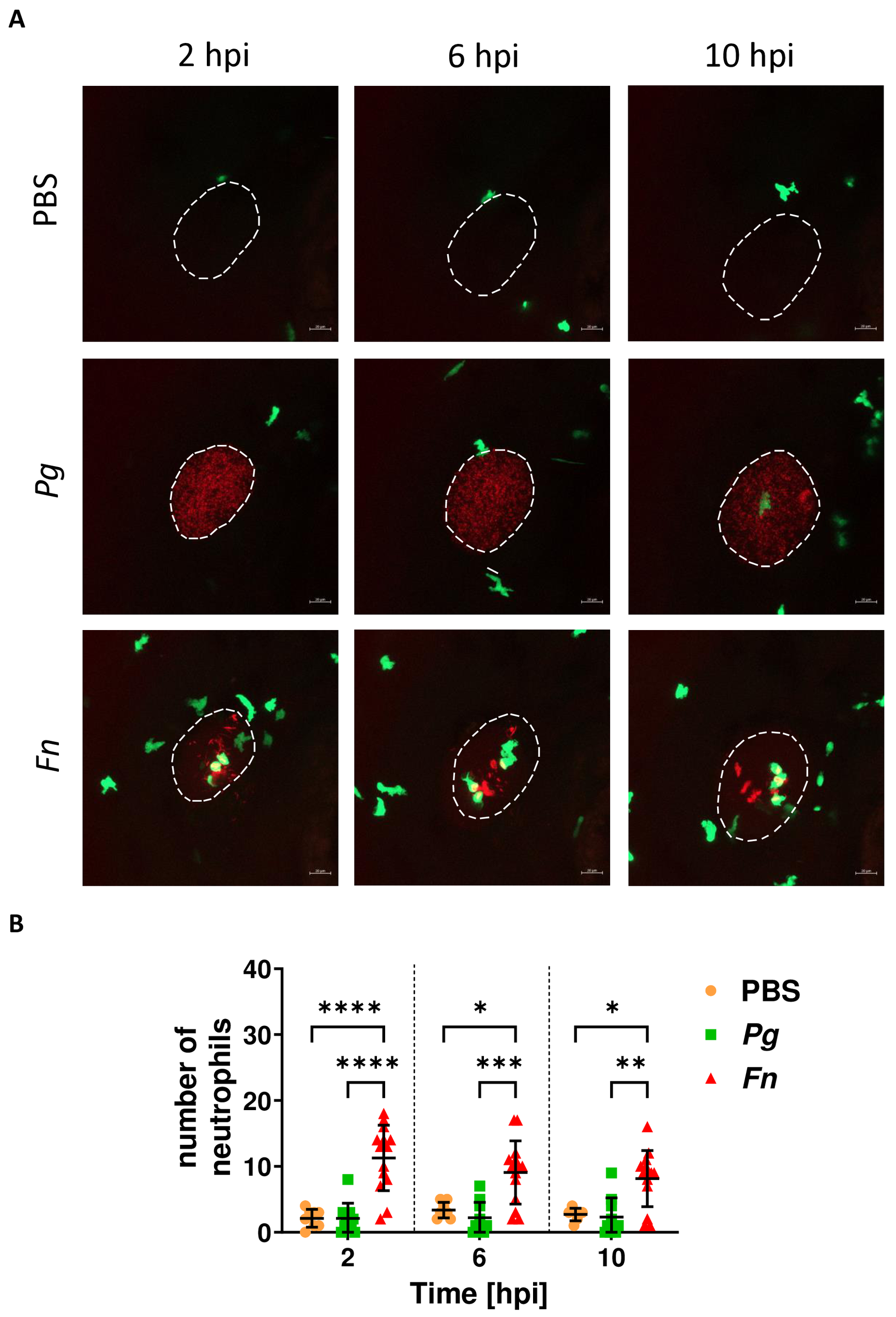

Supplement: S5 Fig — Zebrafish larvae (30 hpf) were infected locally into the otic vesicle with Alexa Fluor 647–SE labelled Pg or Fn and real-time examination of neutrophil migration was performed using transgenic zebrafish larvae Tg(mpx:EGFP). PBS was injected as a control. (A) Representative images of the otic vesicle at 2, 6 or 10 hpi. Scale bar = 20 μm. White dashed lines indicate otic vesicle location. (B) Quantified number of neutrophils attracted toward the infection side at 2, 6 and 10 hpi. Each dot represents quantified data from a single larva obtained in 2 independent experiments. Graphs show means ± SD. Differences between groups were analysed by Two-Way Anova. *p ≤ 0.05, **p ≤ 0.01, *** p ≤ 0.001, ****p ≤ 0.0001. hpi-hours post infection. (TIF) [file ppat.1012821.s005.tif]
